# On the Substitution of Zinc for Lead in the Preparation of Diachylon Plaster

**Published:** 1855-08

**Authors:** N. Gueneau de Mussy


On the Substitution of Zinc for Lead in the Preparation of Diachylon Plaster. By M. N. Gueneau de Mussy. (Bulletin Gén. de Thérap., 1854, p. 569.)—During a stay at the thermal springs of the Pryénees, Dr. de Mussy was struck with the fact that where the baths were used by persons employing diachylon plaster, all those parts of the skin which had been in contact with it became covered with a thick layer of sulphuret of lead, which was very difficult of removal, and he was led to inquire how far it was prudent to maintain these saturnine compounds for a long time in contact with large absorbing ulcerated surfaces. He refers to an instance in which, on two different occasions, lead colic was induced by such an application. At the suggestion of this physician, M. Boileau endeavored to form a plaster with a base of zinc instead of lead. A solution of white soap was brought in contact with a solution of sulphate of zinc, an abundant precipitate of oleo-margarate of zinc fell, which, being washed and dried, was combined with the various other substances which enter into the formation of dyachylum; augmenting, however, the proportion of oil and wax in order to preserve a proper consistence to the plaster.—*Brit. and For. Medico-Chir. Review.*

